# Two Different Clinical Presentations of Acute Limb Ischemia Caused by Acute Thrombotic Events in COVID-19

**DOI:** 10.7759/cureus.17916

**Published:** 2021-09-12

**Authors:** Yudistira P Santosa, Angelina Yuwono

**Affiliations:** 1 Department of Internal Medicine, Atma Jaya Catholic University of Indonesia, Jakarta, IDN

**Keywords:** covid-19, thrombosis, acute limb ischemia, hypercoagulable state, arterial thrombosis

## Abstract

Coronavirus disease 2019 (COVID-19) is a global pandemic caused by severe acute respiratory syndrome coronavirus 2 (SARS-CoV-2). Coagulopathy is frequently found in severe cases of COVID-19 and is usually manifested as a prothrombotic state. Hyperinflammation, endotheliitis, and immobilization during illness are hypothesized to play a role. Acute limb ischemia (ALI) is one of the presentations of arterial thrombosis in COVID-19. We present two cases of middle-aged men with COVID-19 infection, who developed ALI. The first patient developed ALI after 16 days from the initial COVID-19 diagnosis, and the second patient was admitted to the emergency ward due to sudden discoloration of his right lower limb, and COVID-19 was diagnosed during the evaluation.

## Introduction

Coronavirus disease 2019 (COVID-19) was declared a pandemic in March 2020 by the World Health Organization (WHO) [1]. This illness is caused by severe acute respiratory syndrome coronavirus 2 (SARS-CoV-2), which binds to angiotensin-converting enzyme 2 (ACE2) receptors, which are located almost all over cells, but mostly in the lungs, heart, veins, and arteries [2]. COVID-19-associated coagulopathy (CAC) has been extensively described recently. COVID-19, particularly in severe illness, may lead to elevated D-dimer levels, increased fibrin degradation products (FDP), and cytokine overproduction, which are related to coagulopathy [2,3].

In arteries, CAC may manifest as acute limb ischemia (ALI), which is associated with a high rate of mortality and morbidity. ALI incidence increases during pandemics [4]. In this report, we present two cases of middle-aged men with COVID-19 who developed ALI during the course of their illness. We believe this report will enhance our awareness of CAC, especially ALI, which may develop in COVID-19 patients.

## Case presentation

Case 1

**Figure 1:**
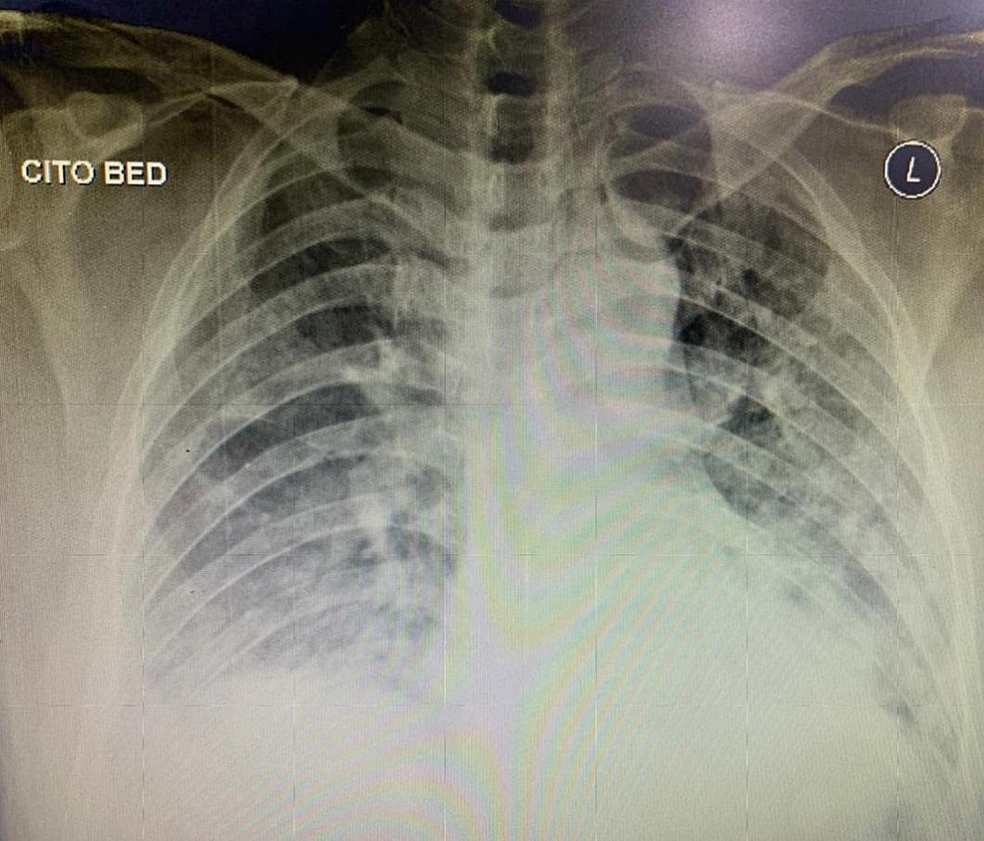
Chest roentgen on admission day showed bilateral infiltrate on both lungs

**Figure 2:**
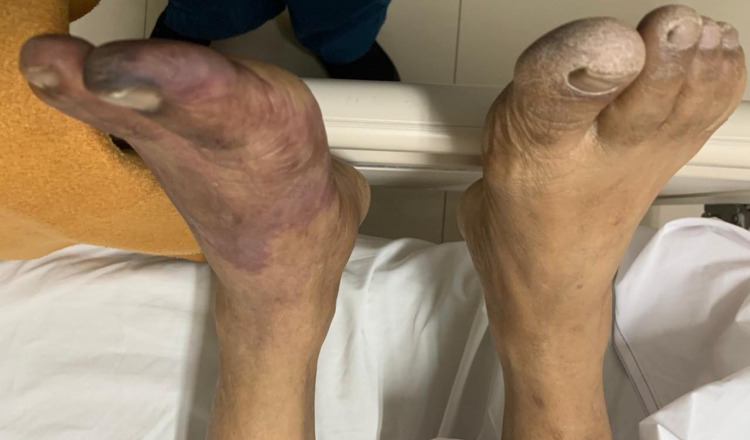
Bluish discoloration appeared on day 16 - image 1

**Figure 3:**
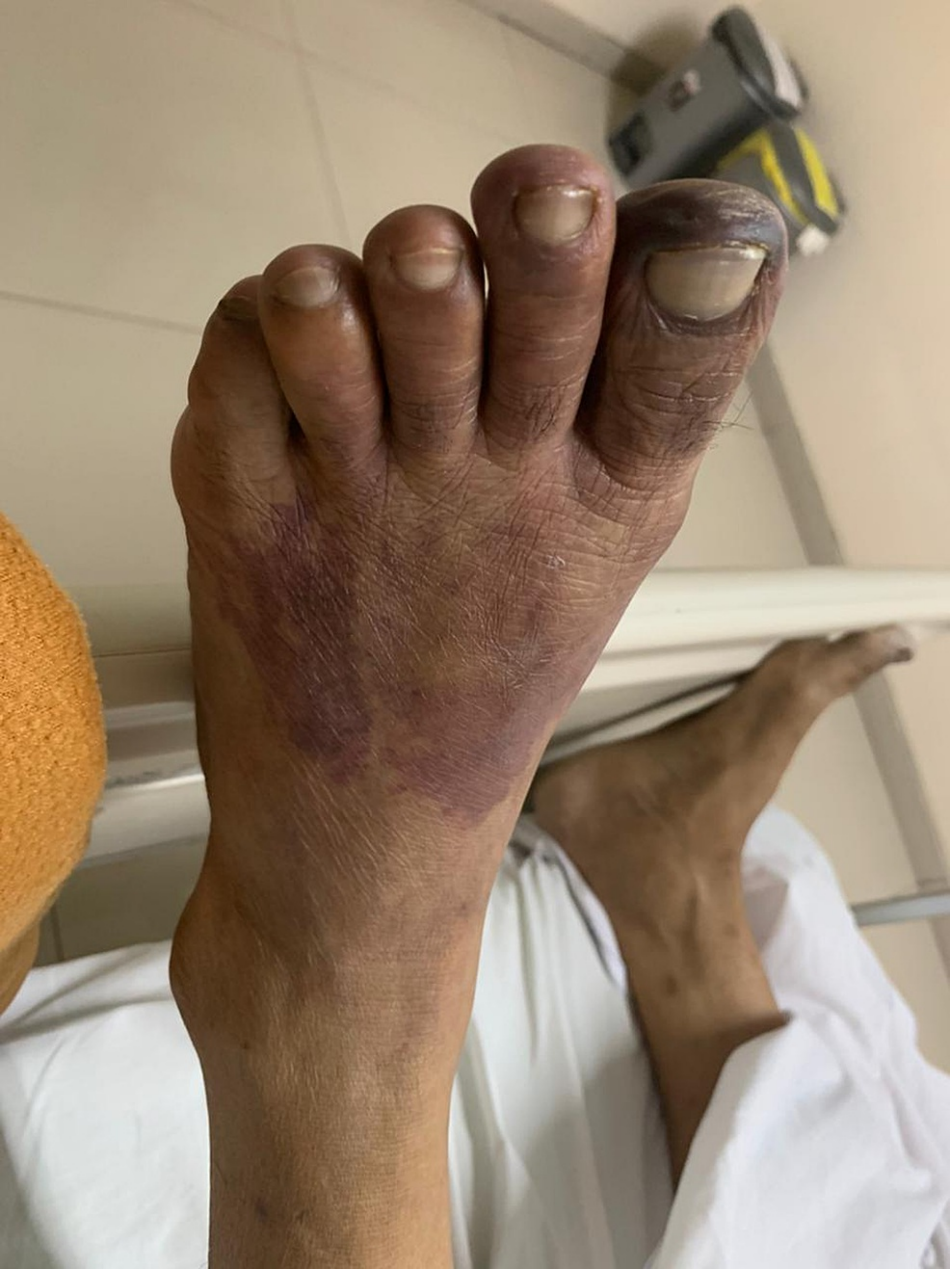
Bluish discoloration appeared on day 16 - image 2

The first case involves a 60-year-old man who developed dyspnea due to severe COVID-19. He was admitted to COVID-19 ICU for 14 days due to respiratory failure. His past medical history was unremarkable and he had had not been on any medication before. He denied any previous surgical history. He also had no family history of cardiovascular and other diseases. He was put on a high-flow nasal cannula. He was lethargic on admission and had elevated respiratory and heart rates. His chest roentgen on admission is shown in Figure 1. His fibrinogen level was 300 mg/dL (normal range: 200-400 mg/dL) and D-dimer was 0.6 mcg/mL (normal range: <0.5 mcg/mL). During his treatment, he was given a prophylactic dose of heparin. His polymerase chain reaction (PCR) swab was negative at the end of the second week, and hence he was transferred to the non-infectious ICU. On day 16, his left foot turned bluish (Figures 2, 3). His D-dimer levels increased to 64 mcg/mL and a Doppler ultrasound revealed total thrombosis on his popliteal artery with no distal flow (Video 1). He also had a secondary bacterial infection during the treatment as a non-ICU patient. He also had severe sepsis due to pneumonia and the condition of his limbs. The patient died before we could manage the limb thrombosis.

**Video 1:** Doppler ultrasound

Case 2

**Figure 4:**
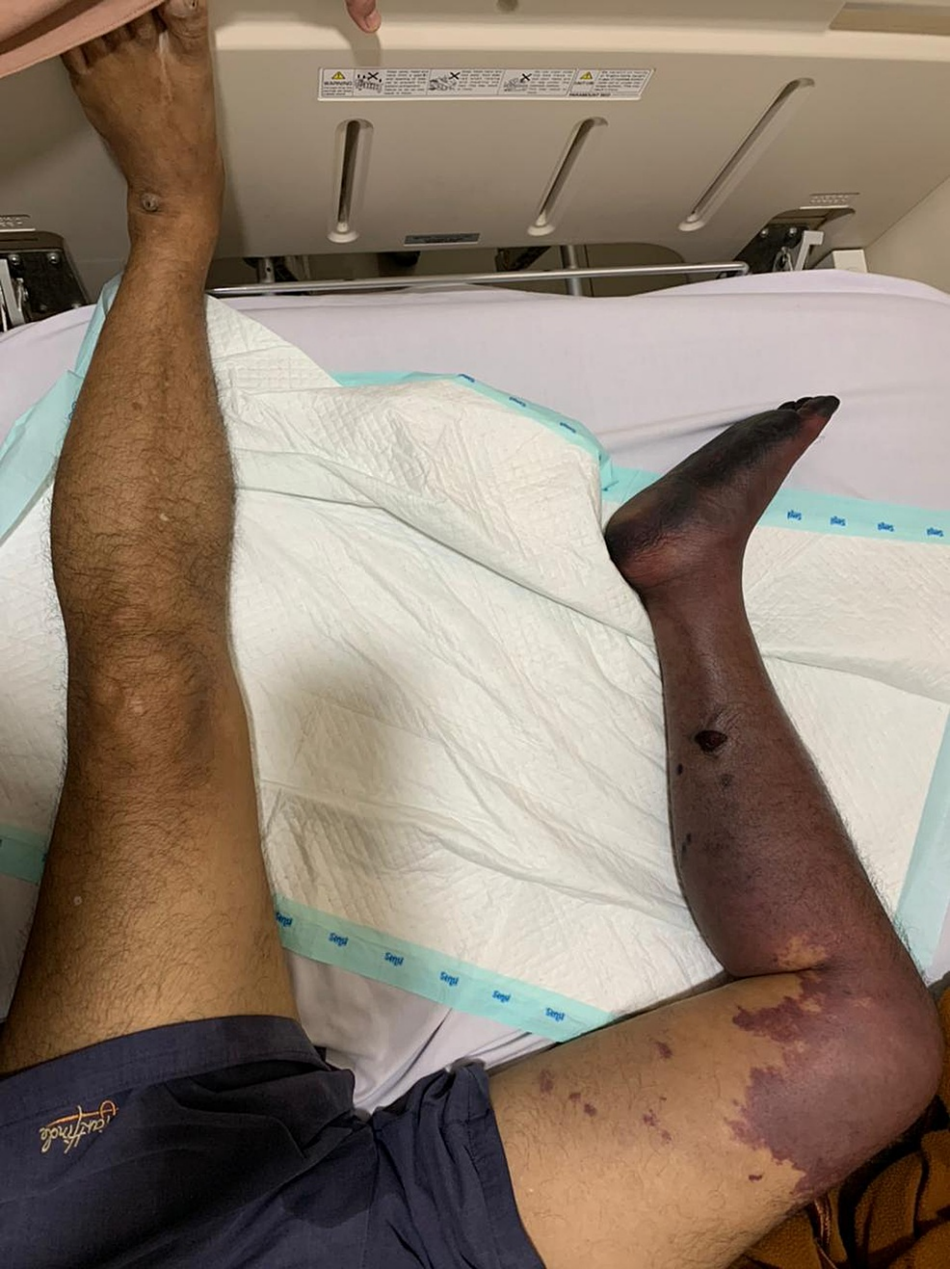
Bluish discoloration from the right distal femur to distal pedis

**Figure 5:**
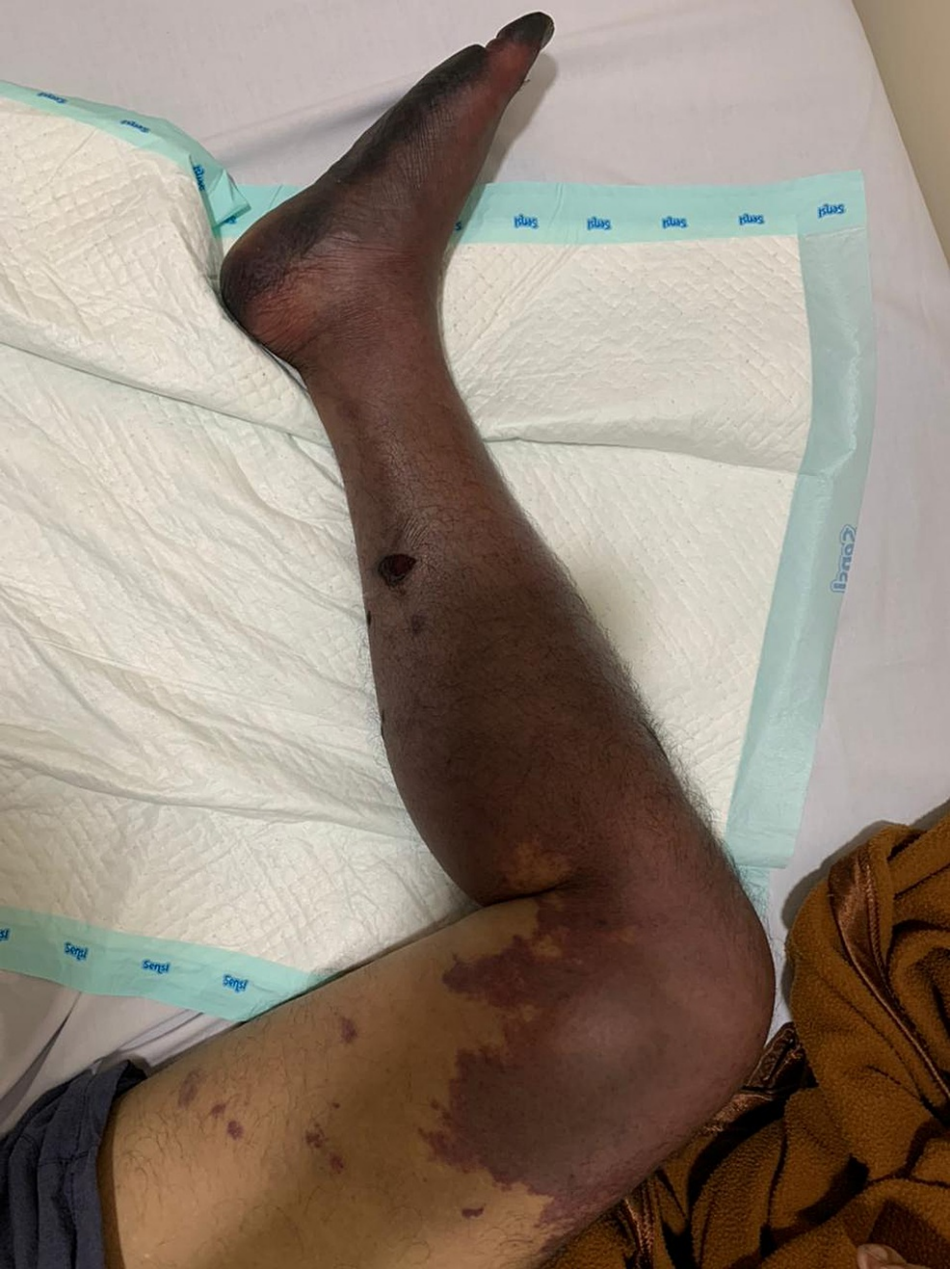
Bluish discoloration from the right distal femur to the foot

**Figure 6:**
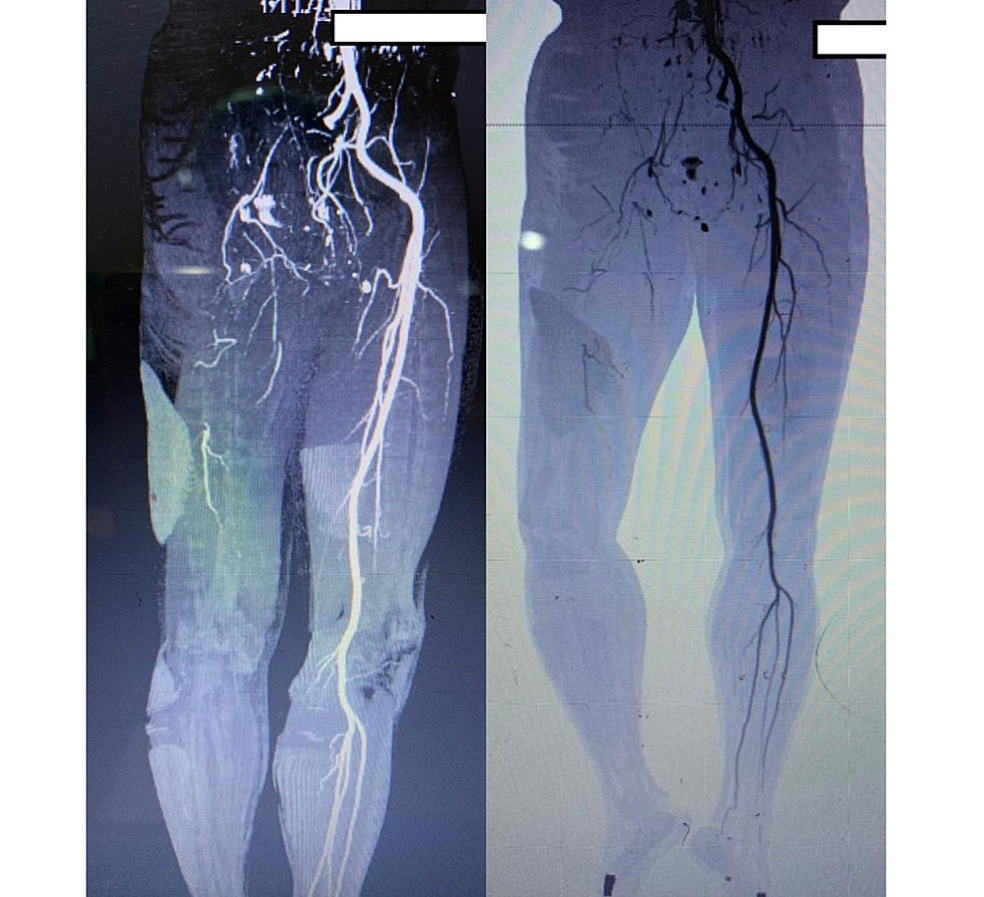
CT angiography of the lower right extremity showed no outflow from the iliac artery CT: computed tomography

A 55-year-old man presented to the emergency department due to sudden bluish discoloration from his right distal femur to distal pedis (Figures 4, 5) and severe pain in his lower right extremity. He had no history of diabetes, hypertension, and renal or other diseases. He also denied any previous medications and surgical history. His family history was unremarkable. He was lethargic and had tachycardia on admission. He also had leukocytosis, an elevated fibrinogen level of 600 mg/dL (normal range: 200-400 mg/dL), and D-dimer level of 7.0 mcg/mL (normal range:<0.5 mcg/mL). A CT angiography of the right lower extremity showed no outflow from the iliac artery. Further evaluation revealed that he had a cough and mild dyspnea; his chest X-ray revealed pneumonia (Figure 6) and the PCR swab was positive. The patient declined the advice to have his right limb amputated and eventually died due to sepsis.

## Discussion

The SARS-CoV-2 infection is associated with multiorgan manifestations. The viruses bind to ACE2 receptors, which are present in all cells, including in endothelial cells, which constitute the lining of the veins and arteries. This promotes viral entry and causes endothelial cell injury, eventually leading to microvascular dysfunction [2]. Upon entering the cells, the virus replicates, and the release of new virus particles results in cell death and infection to other adjacent cells [5]. This process induces organ damage and releases damage-associated molecular patterns (DAMPs) [5,6]. The DAMPs trigger the production of the proinflammatory cytokine, chemokines, and the activation of the complement system, which all lead to the hyperinflammation state known as “cytokine storm” [6-8]. “Cytokine storm” reflects hyperinflammation due to an increase of proinflammatory biomarkers [2,7,9]. This leads to three phases of COVID-19, which are the initial phase (paucisymptomatic), intermediate phase (pneumonia), and hyperinflammation (respiratory failure, vasoplegia, and shock). Clinical deterioration occurs between 7-12 days and is caused by the “cytokine storm” [9]. Severe COVID-19 and obesity are associated with higher levels of c-reactive protein (CRP), ferritin, transaminases, lactate dehydrogenase (LDH), troponin, and acute kidney injury [9]. Increased D-dimer levels and thrombocytopenia in COVID-19 are associated with an increased need for mechanical ventilation, ICU admission, and mortality [10]. In our cases, the first patient presented to the emergency department due to COVID-19 pneumonia; he had respiratory failure and hence he was classified as severe COVID-19 and was admitted to the ICU. On the other hand, the second patient presented with ALI as his chief complaint, and later during the evaluation, he was found to have COVID-19 pneumonia.

CAC has been recently described in COVID-19 patients and is found in 25-55% of severe cases [2,7]. CAC is related to the immunothrombosis process where acute inflammation strongly correlates with a hypercoagulable state [2,10,11]. Interleukin (IL)-6, IL-2, interferon (IFN)-g, tumor necrosis factor (TNF)-a, and neutrophil extracellular traps (NET) play a role in CAC. The inflammatory cytokines interfere with the coagulation and fibrinolysis processes [8]. Endothelial dysfunction in COVID-19 may be due to the direct entry of SARS-CoV-2 to endothelial cells, causing endotheliitis [9,12]. Some studies have shown the presence of antiphospholipid antibodies, which may cause endothelial dysfunction in COVID-19 patients, who manifested cerebral artery obstruction and limb artery obstruction [9,13]. Hypercoagulable state due to inflammation, endothelial cell dysfunction due to endotheliitis, and prolonged immobilization during the illness are consistent with Virchow’s triad [11,12]. Abnormal coagulation may be a sign of clinical decline and has been linked to poor prognosis in previous studies [2,9]. Elevation of D-dimer, fibrinogen, and tissue factor is connected to the activation coagulation cascade and hypercoagulable state, which increase the risk of venous and arterial thrombotic events. Patients with an increased level of D-dimer (three to four times higher than normal) without any symptoms are associated with thrombin generation and complication and are therefore recommended to be admitted [10]. Frequent monitoring of hemostasis parameters leads to early detection of severe disease and the possibility of thrombosis [4,10]. Our first patient had a slight elevation of D-dimer on presentation (0.6 mcg/mL), and the level significantly increased after 16 days of hospitalization (64 mcg/mL), despite being administered with a prophylactic dose of heparin during treatment. Our second patient had increased D-dimer levels on presentation (7 mcg/mL).

Thrombosis occurs more frequently in COVID-19 patients with acute respiratory distress syndrome (ARDS) [14]. Venous thromboembolism, such as deep vein thrombosis (DVT) and pulmonary embolism (PE), occurs more commonly than arterial thromboembolism in COVID-19 [2,9,10,13]. Arterial thromboembolism occurs in 4% of critically ill patients and manifests as ischemic stroke, coronary arterial disease, aortic thrombus, and ALI [13]. In COVID-19 patients, arterial thromboembolism can be unpredictable. Though male gender and the presence of cardiovascular risk factors make patients more susceptible, young people without any risk factors may also have arterial thromboembolism [13]. ALI is caused by a sudden decrease of arterial flow and threatens limb viability. The incidence is increased during the COVID-19 pandemic [12]. Both of our patients were males without any prior medical history and no history of family medical illnesses, yet they developed ALI. It is hypothesized that ALI in COVID-19 may have different pathogenesis and may occur in vessels with no prior atherosclerotic changes [12]. The first patient originally presented with severe COVID-19 and respiratory failure, which increased his risk of having the thrombotic event.

A previous study has shown that 45.5% of COVID-19 patients with ALI presented with acute limb pain as their chief complaint. They had elevated D-dimer levels (69.09%); 67.3% of patients had more than one artery involved. The left and right popliteal artery and left common iliac artery were commonly involved. Arterial thromboembolism may involve upper limbs (obstruction in the brachial and radial artery). Of note, 43.6% of patients with ALI had respiratory failure [13]. Our second patient presented with ALI as his chief complaint and he had elevated D-dimer levels. He also had total arterial obstruction on his right iliac artery. Our second patient was more unique in his disease course. He had total thrombosis on the popliteal artery after 16 days of hospitalization and after his RT-PCR test returned negative. A case report had reported a similar finding where the patient had moderate COVID-19 and developed ALI on the 12th day [12]. Previous case series by Surya et al. has also reported an incidence of ALI occurring one week after a negative RT-PCR test in an 80-year-old woman who had a history of stage I hypertension and dyslipidemia. She had moderate COVID-19 and had been previously hospitalized for 15 days. She had a negative RT-PCR test before being discharged from the hospital, and she returned with ALI due to right popliteal occlusion [15].

ALI is an emergency and is usually treated by early revascularization, double antiplatelet agents, and anticoagulants. The treatment is chosen based on the category of ALI: viable, threatened, or irreversible. Patients who present with irreversible ALI should be advised to have primary amputation [16]. Revascularization in COVID-19-related ALI has lower success rates. This may be related to an ongoing hypercoagulable state during infections. Prolonged use of heparin after revascularization and thrombolytic therapy as adjunctive treatment have shown promise in improving outcomes [4,12,13]. Thrombolytic therapy improves distal circulation, while heparinization prevents the recurrence of distal and proximal thrombosis [4]. Unfortunately, we did not have the opportunity to manage the patients' thrombosis. The first patient died due to severe sepsis that was caused by secondary bacterial pneumonia. The second patient declined to have his limb amputated and later died because of sepsis.

Thromboprophylaxis with a prophylactic dose of anticoagulant is now recommended in all COVID-19 patients who are hospitalized. Low-weight molecular heparin (LMWH) and unfractionated heparin are the usually recommended anticoagulants in COVID-19 [2]. LMWH has anti-inflammatory effects, which is highly beneficial. If administered in the earlier phase of the disease course, LMWH helps reduce inflammation and decrease thrombosis risk. The use of direct oral anticoagulants is still a matter of controversy, as they may be interfered with by antiviral agents [2]. Thromboembolic events in COVID-19 may occur in patients who are receiving a prophylactic dose of anticoagulants, due to an ongoing hypercoagulable state. The prophylactic dose is assumed to be insufficient in countering the prothrombotic state [12,13]. Our first patient's situation may be a case in point, in that he had been given a prophylactic dose of heparin during his severe COVID-19 treatment but the ALI still occurred. Some studies have reported incidences where increasing anticoagulant dose from prophylactic to therapeutic dose resulted in a significant decrease of D-dimer and fibrinogen levels, which was associated with better outcomes [12,13]. The efficacy of switching intravenous heparin or subcutaneous LMWH to oral anticoagulants, after the patient is discharged, is still being evaluated. Patients with D-dimer levels two times the upper limit of normal are at an increased risk of thromboembolism, and oral anticoagulant should be considered to be given until two weeks post-discharge in patients with low bleeding risk [2,13]. However, further studies are still required to evaluate its efficacy.

## Conclusions

COVID-19 is associated with hyperinflammation and hypercoagulable state, which increase the risk of thromboembolic events. ALI may occur as a manifestation of arterial thromboembolism in COVID-19 patients during the course of illness, even in those without any cardiovascular risk factors. This thromboembolic event is related to a poor prognosis. Patients receiving prophylactic doses of anticoagulants may also develop COVID-19-related thromboembolism. Revascularization in COVID-19-related ALI has a low success rate and prolonged administering of heparin after the intervention is recommended to improve outcomes.

## References

[1] Cucinotta D, Vanelli M (2020). WHO declares COVID-19 a pandemic. Acta Biomed.

[2] Becker RC (2020). COVID-19 update: Covid-19-associated coagulopathy. J Thromb Thrombolysis.

[3] Hasan SA, Haque A, Nazir F (2020). Acute limb ischemia: a rare complication of COVID-19. Cureus.

[4] Bellosta R, Luzzani L, Natalini G (2020). Acute limb ischemia in patients with COVID-19 pneumonia. J Vasc Surg.

[5] Parasher A (2021). COVID-19: current understanding of its pathophysiology, clinical presentation and treatment. Postgrad Med J.

[6] Moutchia J, Pokharel P, Kerri A, McGaw K, Uchai S, Nji M, Goodman M (2020). Clinical laboratory parameters associated with severe or critical novel coronavirus disease 2019 (COVID-19): a systematic review and meta-analysis. PLoS One.

[7] Franchini M, Marano G, Cruciani M (2020). COVID-19-associated coagulopathy. Diagnosis (Berl).

[8] Loo J, Spittle DA, Newnham M (2021). COVID-19, immunothrombosis and venous thromboembolism: biological mechanisms. Thorax.

[9] Mezalek ZT, Khibri H, Ammouri W (2020). COVID-19 associated coagulopathy and thrombotic complications. Clin Appl Thromb Hemost.

[10] Gómez-Mesa JE, Galindo-Coral S, Montes MC, Muñoz Martin AJ (2021). Thrombosis and coagulopathy in COVID-19. Curr Probl Cardiol.

[11] Szklanna PB, Altaie H, Comer SP (2021). Routine hematological parameters may be predictors of COVID-19 severity. Front Med (Lausanne).

[12] Topcu AC, Ariturk C, Yilmaz E (2021). Acute limb ischemia in a COVID-19 patient. Thromb Update.

[13] Kariyanna PT, Jayarangaiah A, Kaur A (2021). COVID-19 and acute limb ischemia: a systematic review. Am J Med Case Reports.

[14] Indes JE, Koleilat I, Hatch AN (2021). Early experience with arterial thromboembolic complications in patients with COVID-19. J Vasc Surg.

[15] Surya SP, Santoso RM (2021). A clinical case series of COVID-19-associated acute limb ischemia: real-world situation. Egypt Heart J.

[16] Gerhard-Herman MD, Gornik HL, Barrett C (2017). 2016 AHA/ACC Guideline on the Management of Patients With Lower Extremity Peripheral Artery Disease: Executive Summary: A Report of the American College of Cardiology/American Heart Association Task Force on Clinical Practice Guidelines. Circulation.

